# Withaferin-A suppress AKT induced tumor growth in colorectal cancer cells

**DOI:** 10.18632/oncotarget.7351

**Published:** 2016-02-12

**Authors:** Suman Suman, Trinath P. Das, Suman Sirimulla, Houda Alatassi, Murali K. Ankem, Chendil Damodaran

**Affiliations:** ^1^ Department of Urology, University of Louisville, Louisville, KY 40202, USA; ^2^ Department of Basic Sciences, St. Louis College of Pharmacy, St. Louis, MO 63110, USA; ^3^ Department of Pathology, University of Louisville, Louisville, KY 40202, USA

**Keywords:** tumor growth, EMT, chemoprevention, natural compound, proliferation

## Abstract

The oncogenic activation of AKT gene has emerged as a key determinant of the aggressiveness of colorectal cancer (CRC); hence, research has focused on targeting AKT signaling for the treatment of advanced stages of CRC. In this study, we explored the anti-tumorigenic effects of withaferin A (WA) on CRC cells overexpressing AKT in preclinical (*in vitro* and *in vivo*) models. Our results indicated that WA, a natural compound, resulted in significant inhibition of AKT activity and led to the inhibition of cell proliferation, migration and invasion by downregulating the epithelial to mesenchymal transition (EMT) markers in CRC cells overexpressing AKT. The oral administration of WA significantly suppressed AKT-induced aggressive tumor growth in a xenograft model. Molecular analysis revealed that the decreased expression of AKT and its downstream pro-survival signaling molecules may be responsible for tumor inhibition. Further, significant inhibition of some important EMT markers, i.e., Snail, Slug, β-catenin and vimentin, was observed in WA-treated human CRC cells overexpressing AKT. Significant inhibition of micro-vessel formation and the length of vessels were evident in WA-treated tumors, which correlated with a low expression of the angiogenic marker RETIC. In conclusion, the present study emphasizes the crucial role of AKT activation in inducing cell proliferation, angiogenesis and EMT in CRC cells and suggests that WA may overcome AKT-induced cell proliferation and tumor growth in CRC.

## INTRODUCTION

Despite recent advances in early detection and treatment, colorectal cancer (CRC) patients face high mortality rates [1–3] because some patients develop metachronous tumors, for which limited treatment options are available [4–6]. The survival rate for patients with metastatic CRC is less than 10%. The primary treatments include 5-fluorouracil (5-FU), radiation, and surgery; however, despite treatment, most metastatic cancers eventually cause death [7]. Developing approaches to prevent the progression to metastasis is therefore an important part of a therapeutic strategy, especially for high-risk patients, including those patients already diagnosed with high grade polyps or localized colon cancer [8].

Several signal transduction pathways are known to regulate epithelial-mesenchymal transition (EMT) [9–12]. Among these pathways, the oncogenic activation of AKT has emerged as a central feature [13–18]. The function of AKT is to transduce signals that are responsible for cellular proliferation, survival, growth, angiogenesis, migration, and EMT in CRC as well as in other cancer types [17, 19–23]. Previously, we reported that AKT activation is a key molecular step for EMT, initiating the metastasis process by activating β-catenin, Snail and Slug and simultaneously down-regulating E-cadherin expression in CRC cells [24]. The activation of AKT has been correlated with disease progression in many tumor types, including CRC [25].

The hallmark of EMT is decreased expression of E-cadherin, a cell–cell adhesion molecule thought to repress invasion and metastasis. Activated AKT negatively regulates the transcription of E-cadherin by activating Snail, which then binds to and represses the E-cadherin promoter [15]. Additionally, AKT signaling positively regulates β-catenin–dependent transcription, which is known to participate in CRC development, invasion, angiogenesis, and metastasis [26, 27]. AKT is also known to impart drug resistance to cancers [28]. Recent studies have suggested that the activation of AKT signaling also initiates epigenetic silencing by causing global alterations of histone modifications, specifically the trimethylation of histone H3 lysine 27 (H3K27me3) in breast cancer cells [29], and this alteration is critical for the differentiation of stem and progenitor cells [30].

The important action of AKT is to induce the pro-survival function that causes resistance to apoptosis by upregulating the GSK-3β/mTOR/NF-κB signaling axis in colon cancer [31, 32]. NF-κB is sequestered in the cytosol in a complex comprised of p50/p52, p65, and inhibitory kappa B (IκB). Upon activation of AKT, IκB kinase (IKK) is phosphorylated and degraded, and the NF-κB complex translocates to the nucleus and regulates downstream events [33] that promote the expression of pro-survival genes. Ultimately these events regulate cell proliferation, differentiation, and malignant transformation during cancer progression [34–36]; or transcriptionally inhibit pro-apoptotic genes [37].

Natural compounds represent a major source of current chemotherapeutic agents, and studies have demonstrated both the chemotherapeutic and chemopreventive effects of such substances in preclinical and clinical studies. Withaferin A (WA), an herbal dietary agent that is abundant in the Winter Cherry, *Withania somnifera,* has been used historically in oriental medicine for the treatment of cancers, inflammation, and neurological disorders [38, 39].

The aim of the present study is to determine whether WA overcomes AKT-mediated resistance and inhibits the EMT phenotype using *in vitro* and *in vivo* models of CRC. Our results suggest that oral administration of WA effectively suppresses AKT-induced tumor growth and molecularly inhibits AKT-mediated EMT signaling in colon cancer.

## RESULTS

### AKT accumulates in CRC specimens

**Figure 1:**
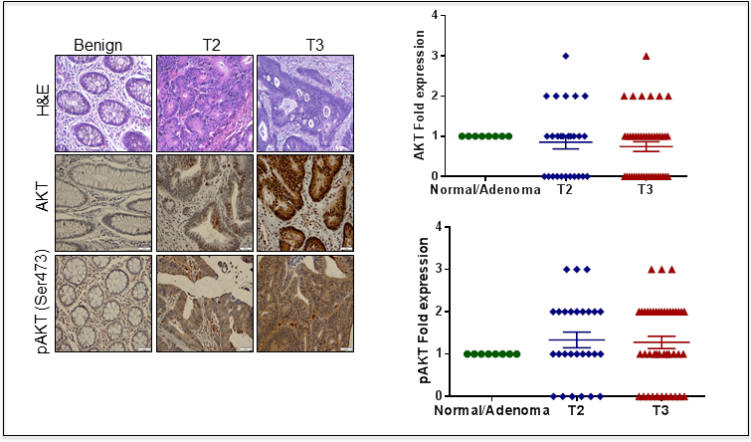
Histological analysis of CRC human tissue microarray (TMA) (**A**) H & E in normal and different stages of CRC. (**B**) Immunohistochemical analysis of AKT phosphorylated AKT ^(Ser 473)^ in normal and different stages of CRC. (**C**) Scatter plot representing AKT phosphorylated AKT ^(Ser 473)^ expression in CRC patients. The data are represented as mean with SEM.

The tissue microarray screening studies indicated that AKT accumulated in tissues from CRC patients in a stage-specific manner that also corresponded to pAKT (ser473) expression (Figure 1). Of the stage T2 and T3 specimens, 84% and 75%, respectively, were positive for pAKT. Further, of these specimens, 57% and 62.5%, respectively, showed more than a 2–3 fold higher expression of pAKT compared with the benign patient samples (Figure 1).

### WA inhibits AKT-induced cell proliferation in CRC cells

**Figure 2:**
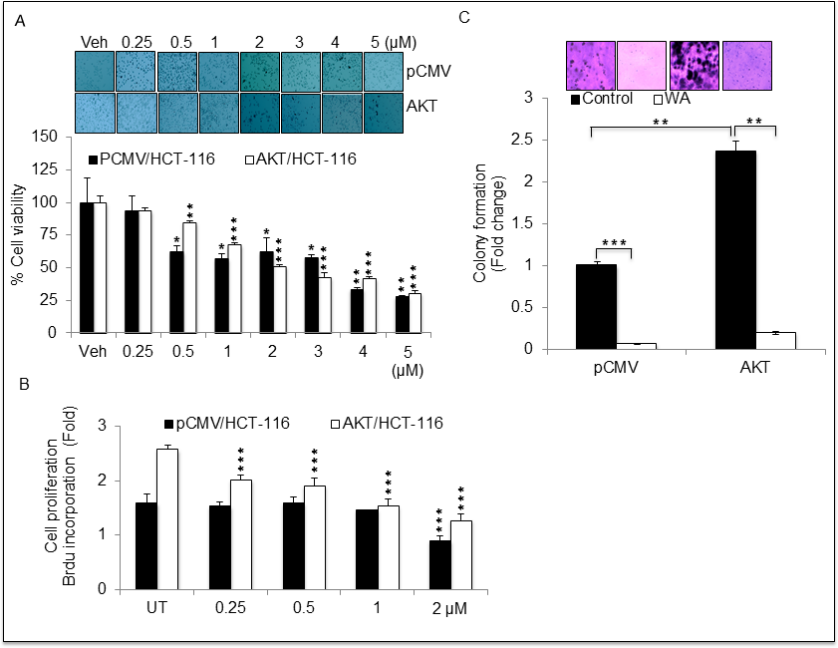
Withaferin A (WA) inhibits cell growth in HCT-116 cells stably expressing pCMV and AKT (**A**) HCT-116 stable transfactants were treated with indicated concentration of WA or Vehicle (DMSO) for 24 h followed by MTT assay for cell viability. (**B**) Cell proliferation was assessed by BrdU incorporation-based cell proliferation assay in HCT-116 stable transfactants treated with indicated concentrations of WA. (**C**) Anchorage independent growth was assessed by soft agar colony formation assay in HCT-116 stable transfactants. Data are presented as the mean ± standard deviation (SEM/SD) of three independent experiments. **P < 0.005, and ***P < 0.0001.

To determine whether WA inhibits AKT-induced cell proliferation in CRC cells, we measured cell proliferation for cells expressing pCMV/HCT-116 or AKT/HCT-116 using MTT, trypan blue and BrdU assays. Treatment with WA for 24 h inhibited the growth of HCT-116 transfectants (pCMV and AKT) in a dose-dependent manner (Figure 2A and 2B). Similar results were found in trypan blue assays (data not shown). Furthermore, the WA potency was confirmed with colony-forming assays. As expected, colon cancer cells overexpressing AKT exhibited more colonies than the cells transfected with the vector alone. WA significantly inhibited AKT-induced colony formation in AKT/HCT-116 cells as well as pCMV/HCT-116 cells (Figure 2C). Collectively, these results suggest WA may be a potent molecule with the potential to overcome AKT-induced cell proliferation in CRC cells.

### WA inhibits AKT/mTOR/NF-κB signaling axis in HCT-116 transfectants

**Figure 3:**
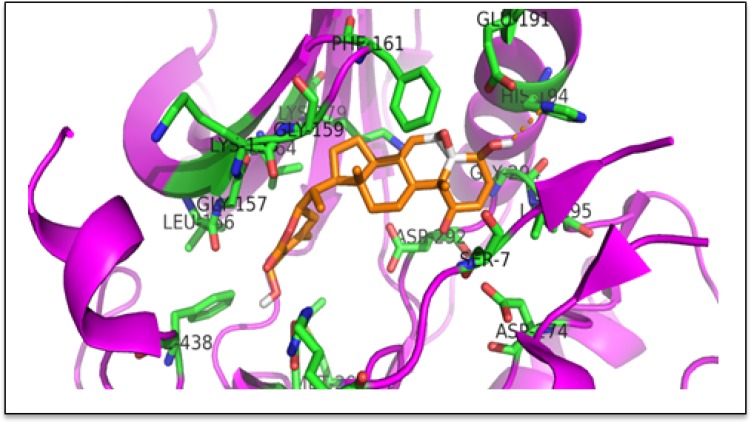
Binding pose of WA in the binding pocket of AKT kinase domain (PDB ID: 3OCB) The ligand (WA) is shown as an orange stick model; the protein is shown as a cartoon and the residues within 4 Ångstroms of a ligand are shown as green stick models. The hydroxyl group of WA is forming a hydrogen bond with the sidechain of His 194. The figure was prepared with PYMOL.

**Figure 4:**
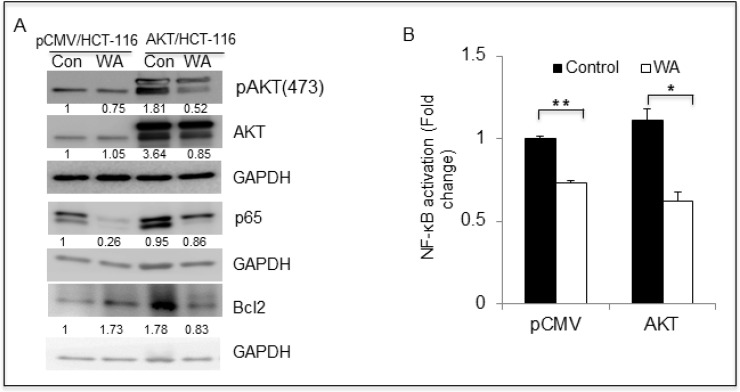
WA inhibits AKT cell signaling in colon cancer cells (**A**) HCT-116 transfectants were treated with WA and vehicle (DMSO) for 48 h, followed by whole-cell lysate preparation. The lysates were quantitated and equal amounts of proteins were loaded for western blot analysis. (**B**) NFκB binding activity was measured in nuclear protein fractions of HCT-116 stable transfactants treated with WA.

The results described above demonstrated that AKT promotes cell growth and that WA effectively inhibited such AKT-mediated growth in pCMV/HCT-116 and AKT/HCT-116 CRC cells. Therefore, we sought to determine whether WA binds to AKT via docking studies. The published crystal structure of the AKT1 kinase domain (PDB ID: 3OCB) was used for the docking studies. The best docking pose of WA in AKT-1 has an affinity of −11.0 kcal/mol. The amino acid residues of AKT-1 within 4 Ångstroms of the best docking pose of WA include ARG-4, SER-7, LEU-156, GLY-157, LYS-158, GLY-159, PHE-161, VAL-164, LYS-179, GLU-191, HIS-194, GLU-234, ASP-274, MET-281, ASP-292, GLY-294, LEU-295, and PHE-438. It appears that the hydroxyl group of WA forms a hydrogen bond with the nitrogen on the side chain of HIS-194. The results suggest that WA binds to AKT1 with a strong affinity (Figure 3). Next, we examined whether WA inhibits the AKT signaling network in CRC cells. As seen in Figure 4A, WA inhibited AKT activation by downregulating AKT-phosphorylation (Ser473) in both AKT/HCT-116 and pCMV/HCT-116 cells. Many groups have demonstrated that AKT phosphorylates RelA, a major subunit of NF-κB, through IKK [41] therefore, we explored whether AKT inhibition affects NF-κB expression in HCT-116 cells. As shown in Figure 4B, WA treatment downregulated endogenous as well as AKT-induced NF-κB activation in AKT/HCT-116 cells.

### WA overrides AKT-induced EMT in CRC cells

**Figure 5:**
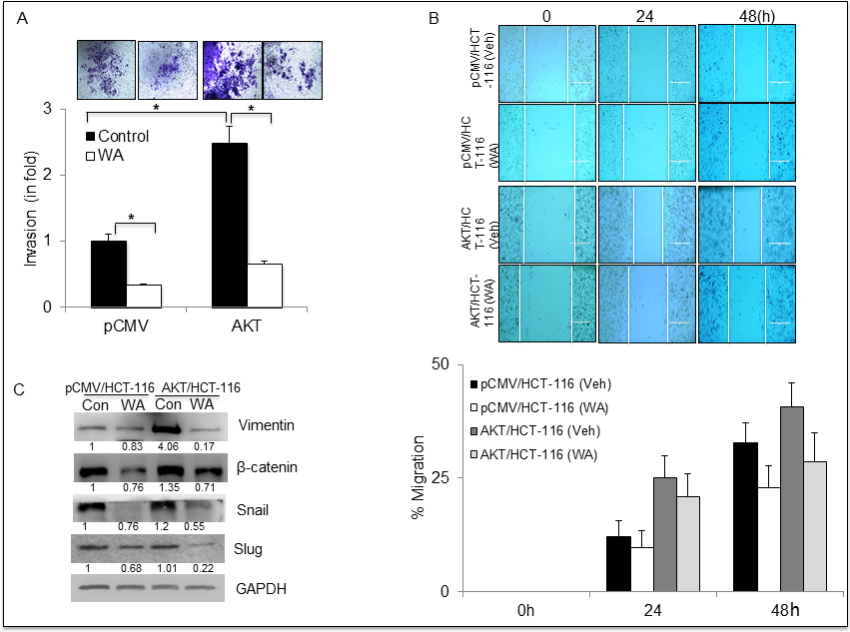
WA inhibits EMT in HCT-116 stable transfectants (**A**) Transwell invasion assay performed using Boyden chambers. The invaded cells were stained with crystal violet and counted. (**B**) HCT-116 transfectants were treated with WA and vehicle (DMSO) followed by whole-cell lysate preparation. The lysates were quantitated and equal amounts of proteins were loaded for western blot analysis for EMT markers. (**C**) For the wound healing assay, a wound was created and the cells were treated with WA or Vehicle (DMSO). The wound gap was photographed at the same points using EVOS microscope and the distance between the two edges of wound was measured using NIS-Element software. Data are expressed as mean ± SD of three independent experiments. *P < 0.05.

Earlier, we established that AKT regulates EMT in CRC cells by altering the expression of EMT markers. To determine the anti-EMT role of WA on pCMV/AKT transfectants, invasion assays were performed on AKT/HCT-116 and pCMV/HCT-116 cells treated with WA. WA significantly inhibited the invasion potency of AKT/HCT-116 (2.5-fold) and pCMV/HCT-116 cells, as shown in Figure 5A. Similarly, the relative migration rates were reduced in WA-treated AKT/HCT-116 and pCMV/HCT-116 cells (Figure 5B). Further, Western blot analysis confirmed that WA downregulated the expression of β-catenin, vimentin, Snail and Slug in both AKT/HCT-116 and pCMV/HCT-116 cells (Figure 5C).

### WA induces apoptosis in CRC cells

**Figure 6:**
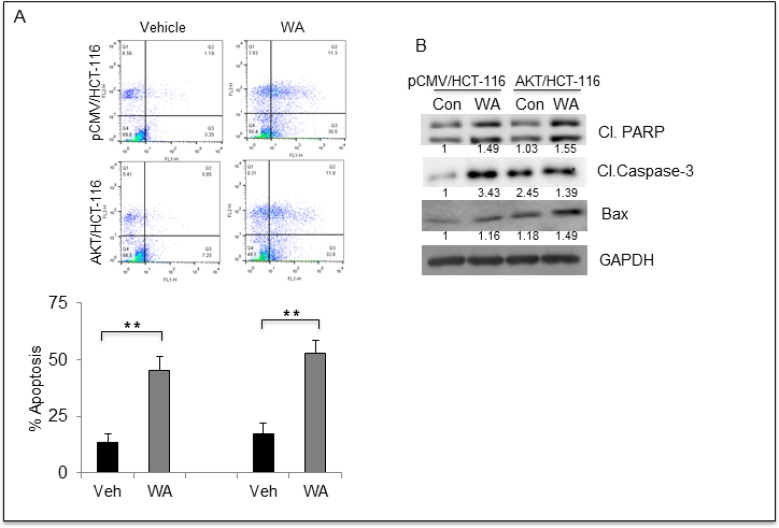
WA induces apoptosis in HCT-116 transfectants (**A**) Flow cytometry based apoptosis assay was performed using Annexin V –FITC and Propidium Iodide staining for HCT-116 transfectants treated with WA. (**B**) HCT-116 transfectants were treated with WA and vehicle (DMSO) followed by whole-cell lysate preparation. The lysates were quantitated and equal amounts of proteins were loaded for western blot analysis for apoptosis markers.

To better understand the mechanism involved in WA-mediated cell growth inhibition, we performed apoptosis assays, and interestingly WA induced apoptosis in both AKT/HCT-116 and pCMV/HCT-116 cells (Figure 6A). These results were confirmed at the molecular level by Western blot analysis. The Western blot data showed an increase in classical apoptosis markers such as cleaved PARP, cleaved caspase-3 and BAX in response to WA treatment (Figure 6B). These results clearly indicate that WA induces apoptosis in CRC cells.

### Oral administration of WA inhibits the aggressive tumor growth of CRC cells overexpressing AKT

**Figure 7:**
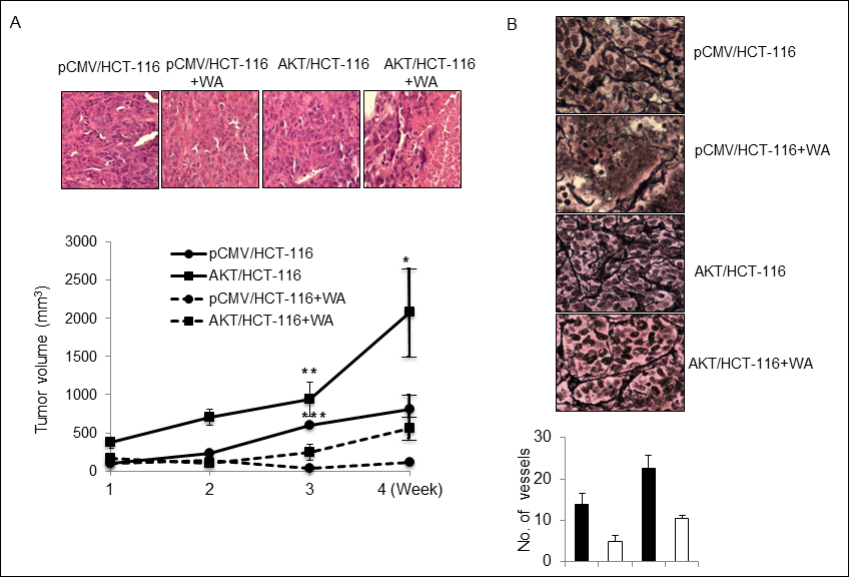
WA inhibits *in vivo* tumor growth (**A**) Nude mice were injected subcutaneously with HCT-116 stable transfactants. Tumor xenografts were monitored twice weekly. Control and treatment group mice were orally gavaged with oil and WA (5 mg/kg body weight) respectively for four weeks. The graph represents the tumor volumes in mm^3^. (**B**) WA inhibits the microvessel formation, as depicted by immunohistochemical analysis for RETIC staining in HCT-116 stable transfectant tumor xenografts. *P < 0.05, **P < 0.005, and ***P < 0.0001.

We demonstrated previously that AKT/HCT-116 xenografts are highly aggressive compared with pCMV/HCT-116 xenografts [24]. Therefore, we next determined the *in vivo* efficacy of WA in animals xenografted with pCMV/HCT-116 and AKT/HCT-116 cells. Following oral administration of WA five days/week for four weeks, an analysis of the tumor volumes revealed that WA treatment significantly inhibited the growth of both pCMV/HCT-116- and AKT/HCT-116-induced CRC tumors, without causing significant toxicity to the animals (Figure 7A). Further, we assessed the micro-vessel formation in AKT/HCT-116 and pCMV/HCT-116 tumor xenografts. The IHC results for RETIC, an angiogenic marker, clearly showed inhibition of micro-vessel formation in WA-treated AKT/HCT-116- and pCMV/HCT-116-induced tumors (Figure 7B).

## DISCUSSION

Our results confirmed that the activation of AKT regulates many signaling pathways, including those necessary for cell survival, proliferation and EMT of CRC. We also demonstrated that WA over rides AKT-induced growth in both *in vitro* and *in vivo* models of CRC.

Aberrant expression of AKT has been documented in many cancer types [31, 42], and our clinical data confirmed both AKT activation (pAKT^ser 473^) and accumulation in a stage-specific manner in human CRC tissues. A number of studies have suggested the involvement of AKT signaling in different types of human metastatic cancers and its potential role in chemoresistance [43–45]. Several preclinical studies have also shown that the inhibition of AKT reduces cell proliferation, migration and EMT, suggesting that such an approach may be useful as a treatment for many cancer types, including colon cancer. As a result, several groups are now developing small molecules that can inhibit AKT activation. For example, pharmacological inhibitors such as LY294002 and wortmannin (PI3K inhibitor) have been shown to inhibit cell proliferation in both *in vitro* and *in vivo* studies [46, 47]; however, toxicity issues have impeded their clinical usage. Recently, perifosine, an oral inhibitor of AKT, has been tested in a phase-III clinical trial for the treatment of metastatic CRC [48]. Similarly, rapamycin, an inhibitor of mTOR signaling, has been successfully used in both preclinical and clinical trials; however, rapamycin specifically targets mTOR1, and feedback activation of AKT limits its clinical usage [49].

In our study, WA significantly inhibited phosphorylated-AKT (pAKT) without downregulating total AKT expression. Natural compounds, such as quercetin and brassinin, have been shown to inhibit AKT activity in many cancer cell types, including CRC [50], [51]. Earlier, we reported that WA downregulated AKT/NOTCH signaling and simultaneously induced apoptosis in a panel of colon cancer cell lines [40]. Our current docking studies suggest that in AKT/HCT-116 cells, WA binds to AKT and inhibits the phosphorylation of AKT, resulting in the inhibition of many phenotypic cascades, such as cell proliferation, invasion, migration and tumor growth.

We and others have demonstrated [13] that AKT plays a critical role in EMT, the pathway by which epithelial cancer cells initiate metastasis. In the current study, HCT-116 cells overexpressing AKT showed increased expression of β-catenin, Snail and Slug as detected by Western blot analysis; however, WA treatment led to the downregulation of mesenchymal markers and ultimately the inhibition of EMT. Prior studies have suggested that TGF-β activation increases the expression of Snail family proteins and decreases the expression of E-cadherin, resulting in EMT. In contrast, the inhibition of TGF-β signaling has been demonstrated to downregulate Snail family proteins and increases the expression of E-cadherin, resulting in the epithelial phenotype in human epithelial cells [54].

In our studies, the activation of AKT showed the potential to induce EMT in colon cancer cells, and the inhibition of AKT by WA reversed the EMT phenomena in HCT-116 cells overexpressing AKT. Taken together, these findings suggest that targeting AKT signaling may be an important approach for the treatment of aggressive colon cancer. The attenuation of the expression of the mesenchymal markers β-catenin, Snail, Slug, and vimentin by WA may have alleviated AKT-induced EMT in both the *in vitro* and *in vivo* models. Thus, the present study established the effect of AKT on EMT signaling and suggests that AKT may cause the reversion of the EMT phenotype to an MET phenotype, resulting in less aggressive and less invasive cancer.

In this study, we demonstrated that the activation of AKT plays a major role in triggering EMT in colon cancer cells, suggesting that targeting AKT signaling could lead to the inhibition of EMT and ultimately less invasive cancer. We also found that WA is a potent small molecule that could override AKT-induced cell proliferation and tumor growth of colon cancer cells. Although we did not evaluate the metastasis of tumors overexpressing AKT due to the increase in tumor burden on animals, our results demonstrate that AKT represents an attractive target, and developing either pharmacological agents or derivatives of WA may be a viable therapeutic option for treating patients with metastatic colon cancer.

## MATERIALS AND METHODS

### Cell lines and reagents

The human colorectal cancer cell line HCT-116 was purchased from the American Type Culture Collection (Manassas, VA, USA). HCT-116 cells were grown in DMEM supplemented with 10% fetal bovine serum, 1% l-glutamine, and antibiotics in the presence of 5% CO_2_ at 37°C in an incubator. Human HCT-116 colorectal cancer cells stably overexpressing AKT (AKT/HCT-116), as well as vector-transfected cells (pCMV/HCT-116) [24], were selected and used for these experiments. The cells were grown in DMEM supplemented with 10% fetal bovine serum, 1% l-glutamine, 300 μg/ml of G418 and antibiotics in the presence of 5% CO_2_ at 37°C in an incubator.

### Cell proliferation/soft agar colony formation assay

To determine the anti-proliferative effect of WA, the cancer cells were treated in various concentrations of WA and/or dimethyl sulphoxide (DMSO) for 24 h followed by MTT ((3-[4, 5-Dimethylthiazol-2-yl]-2, 5-diphenyltetrazolium bromide) assay as per the manufacturer's protocol (Sigma Aldrich, Saint Louis, MO, USA). Cell proliferation was quantified for the selected clones stably expressing pCMV/HCT-116 or AKT/HCT-116 using the Bromodeoxyuridine (BrdU) incorporation assay (Cell Signaling, Danvers, MA, USA), according to the manufacturer's protocol, and the trypan blue exclusion method [51]. Anchorage-independent growth was monitored by colony formation assay using a CytoSelect^™^ 96-well *in vitro* tumor sensitivity assay kit (Cell Biolabs, Inc, San Diego CA, USA). The cell harvesting and assay were performed as per the manufacturer's instructions using 5 × 10^3^ cells (pCMV/HCT-116 and AKT/HCT-116) [24].

### Invasion assay

To determine the invasiveness of pCMV/HCT-116 or AKT/HCT-116 cells, invasion assays were performed and evaluated by employing Boyden chambers equipped with polyethylene terephthalate membranes with 8-μm pores (BD Biosciences, San Jose, CA, USA). The cells were cultured in complete medium for 24 h prior to detachment with trypsin EDTA. Subsequently, 5 × 10^4^ cells per chamber were re-suspended in culture medium and layered on the Matrigel, and after an additional 24 h, the invaded cells were counted using an AMG EVOS digital inverted microscope (Life Technologies, Carlsbad, CA, USA) as described earlier [24].

### Wound-healing migration assay

The HCT-116 transfectants were plated in six-well plates and cultured until they reached confluency. A linear wound was gently created in the monolayer of cells with a 200 ul sterile pipette tip. The cells were then washed with PBS/growth medium to remove the detached cells, followed by gently adding fresh medium. The distance between the wound gap was photographed at the same point at 0, 24 and 48 h time points. The wound gap was measured by using NIS-Element AR software (Nikon Instruments Inc, Melville, NY, USA), and the distance between the opposing edges of the wound was measured in micrometers.

### Protein extraction and western blotting

The total protein extracts from the pCMV/HCT-116 and AKT/HCT-116 cells were prepared with Mammalian Protein Extraction Reagent (Thermo Scientific, Rockford, IL, USA) according to the manufacturer's protocol. Western blotting was performed using specific antibodies against Snail, Slug, β-catenin, GAPDH, Vimentin, NF-κB (p65), and actin (Santa Cruz Biotechnologies, Dallas, TX, USA). AKT, pAKT, BAX and BCL-2 were purchased from Cell signaling (Danvers, MA, USA). The positive bands were detected using enhanced chemiluminescence.

### Docking studies

The 3D crystal structure of the AKT1 kinase domain (PDB ID: 3OCB) was downloaded using the protein preparation wizard of the Maestro program (Release 2015-1: Maestro, version 10.1, New York, NY). The downloaded protein structure was preprocessed by assigning bond orders, adding hydrogens, creating disulfide bonds, and filling in missing side chains using prime, cap termini, and deleting waters beyond 5 Ångstroms of het groups. The preprocessed structure was reviewed and further refined by assigning sample water orientations to the H-bonds and removing waters with less than 3 H-bonds to non-waters and minimized using the OPLS_2005 force field. The protein structure prepared from the above procedure was used to generate the docking grid. The receptor was defined by placing the grid box on the centroid of the workspace ligand (XM1) and selected to dock ligands similar in size to the workspace ligand. Extra precision (XP) docking was performed using the glide module by selecting the generated grid file and choosing WA as the ligand.

### NF-κB activation assay

To measure the level of NF-κB activation in pCMV/HCT-116 and AKT/HCT-116 cells, nuclear extracts were prepared using N-PER-Nuclear and Cytoplasmic Extraction Reagents (Thermo Scientific, Rockford, IL, USA) according to the manufacturer's protocol. A TransAM NF-κB family transcription factor assay kit (Active Motif, Carlsbad, CA, USA) was used to detect NF-κB activation according to the manufacturer's protocol.

### Xenograft studies

All of the animals were housed under pathogen-free conditions, and the experiments were performed in accordance with the approval of the institutional Animal Care and Use Committee. Balb/c athymic nude mice (*nu*/*nu*) were purchased from the Jackson Laboratory (Bar Harbor, ME, USA) and used at 6–8 weeks of age. For xenograft studies, pCMV/HCT-116 or AKT/HCT-116 cells (1.5 × 10^6^) in a 50-μl final volume of phosphate-buffered saline (PBS) were injected subcutaneously into separate flanks of the mice (6–8 animals). The mice were monitored twice weekly for tumor growth, and the tumor volumes were measured once per week. WA (5 mg/kg BW) was administered orally five days per week for four weeks.

### Immunohistochemical analysis of xenografts

For the immunohistochemical analysis, the tumor samples derived from the pCMV/HCT-116 and AKT/HCT-116 xenografts were fixed and stained with hematoxylin and eosin (H & E) for examination with light microscopy. Similarly, the tumor sections were subjected to RETIC staining to measure the size of the vessels in the tumor specimens.

### Immunohistochemistry (IHC) analysis of human CRC tissue microarray (TMA)

For the immunohistochemical analysis, a human CRC TMA (Cat no. COC1501) was purchased from Pantomics (Richmond, CA, USA). For each grade, the TNM classification is provided in the product data sheet; 8 normal, 26 stage I, and 43 stage II tumor specimens were included. The CRC tissue array slide was incubated with the primary antibody for AKT and pAKT (ser473), followed by secondary antibody incubation and analysis under a light microscope. Each tissue core was evaluated for pAKT/AKT staining as follows: the staining location was recorded as either cytoplasmic or nuclear; the staining pattern, as either granular or diffuse; and the staining intensity, as weak, moderate or strong. Each tissue core was scored on a scale of 0 to 3+, with pAKT/AKT expression in normal tissue defining the baseline score of 1+. A score of 0 indicates no staining, a score 1+ indicates either granular or weak staining, a score of 2+ indicates moderate diffuse staining, and a score of 3+ indicates strong diffuse staining. The composite score for each tissue core was calculated by multiplying the two values together to normalize the heterogeneity of the staining and multifocal nature of the tumor by two different pathologists.

### Statistical analysis

The data were presented as the mean ± standard deviation (SD or SEM). The significant differences between the groups were determined using the unpaired Student's *t*-test. The significant differences were established at *p* < 0.05. All of the statistical analyses were performed using Prism 6 software (GraphPad Software Inc, La Jolla, CA, USA).
